# Acute bilateral renal infarction after left upper pulmonary lobectomy: a case report

**DOI:** 10.1186/s44215-023-00068-9

**Published:** 2023-09-07

**Authors:** Tomohiko Matsuzaki, Kazuhiro Matsuo, Tomoki Higeta, Kie Shioyama, Kei Nakano, Hiroto Onozawa, Takaaki Tsuboi, Ryo Hashimoto, Atsushi Wada, Naohiro Aruga, Ryota Masuda, Masayuki Iwazaki

**Affiliations:** Division of General Thoracic Surgery, Department of Surgery, Tokai University School of Medicine, Ishehara, Kanagawa Japan

**Keywords:** Lung cancer, Left upper lobectomy, Renal infarction, Case report

## Abstract

**Background:**

Left upper lobectomy (LUL) is considered a risk factor for thrombus formation in the pulmonary vein stump compared with other lobectomies, and it occurs in 11.7–13.5% of cases. Although cerebral infarction after LUL has been reported in many articles, reports of bilateral renal infarction after lobectomy are rare.

**Case presentation:**

A 70-year-old male patient was admitted to our hospital with a diagnosis of lung cancer. The nodule’s diameter was 2.5 cm on computed tomography, and it was diagnosed as a non-small cell lung carcinoma by a bronchoscopic biopsy. The clinical stage was cT1c N0M0 (c-stageIA3). The patient underwent thoracoscopic LUL and systemic lymph node dissection. No complications were observed during the operation, and the immediate postoperative period was uneventful until postoperative day 3. On postoperative day 4, he experienced fever, abdominal pain, and nausea, which spontaneously resolved. Laboratory data showed an elevated white blood cell count, and elevated serum lactate dehydrogenase and creatinine concentrations. Contrast-enhanced computed tomography from the thorax to the pelvic cavity showed a partial defect of the bilateral kidneys. We diagnosed the patient with bilateral renal infarction.

**Conclusions:**

Bilateral renal infarction after lobectomy is a rare and serious condition, which should be diagnosed as early as possible. Since all cases of reported renal infarctions occurred after LUL, special attention should be paid to postoperative management after performing LUL.

## Background

Left upper lobectomy (LUL) is considered to have a higher risk of thrombus in the pulmonary vein stump (PVS) than other lobectomies [1, 2]. Although cerebral infarction after LUL has been reported in many articles [3–5], reports of bilateral renal infarction after lobectomy are rare. We report a rare case of acute bilateral renal infarction after LUL.

## Case presentation

**Fig. 1:**
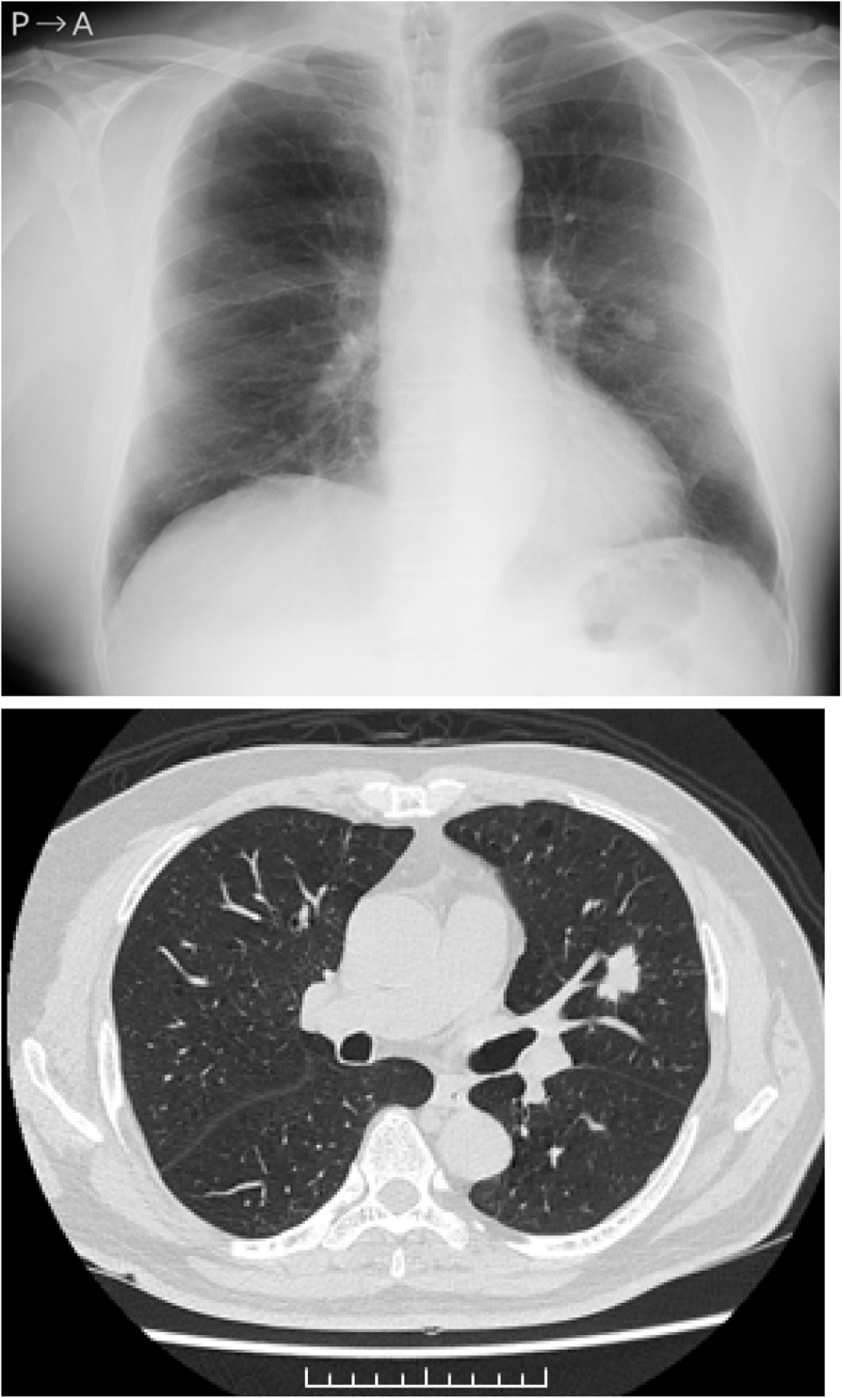
Chest X-ray and computed tomographic images. **a** Chest X-ray film showing a tumor shadow in the left middle field. **b** Chest computed tomography showing a 25 × 13 mm nodule in the left upper lobe

A 70-year-old male patient was admitted to our hospital with the diagnosis of lung cancer. He had a medical history of dyslipidemia and had smoked three packs of cigarettes each day for 50 years. A physical examination and blood test analysis were normal, and cardiac function was excellent. The preoperative electrocardiogram showed no atrial fibrillation, and no thrombus was detected by preoperative echocardiography. Other etiological promotors of renal infarction are unspecified. A chest X-ray showed a nodule in the left middle lung field (Fig. 1a). The nodule’s diameter was 2.5 cm on computed tomography (Fig. 1b), and it was diagnosed as a non-small cell lung carcinoma by bronchoscopic biopsy. Positron emission tomography/computed tomography and head magnetic resonance imaging did not show anything abnormal, except for a mass located in the left upper lobe. The clinical stage was cT1c N0M0 (c-stageIA3).

The patient underwent thoracoscopic LUL and systemic lymph node dissection in our hospital. The operation time was 152 min, and the intraoperative volume of blood loss was 50 ml.

**Fig. 2:**
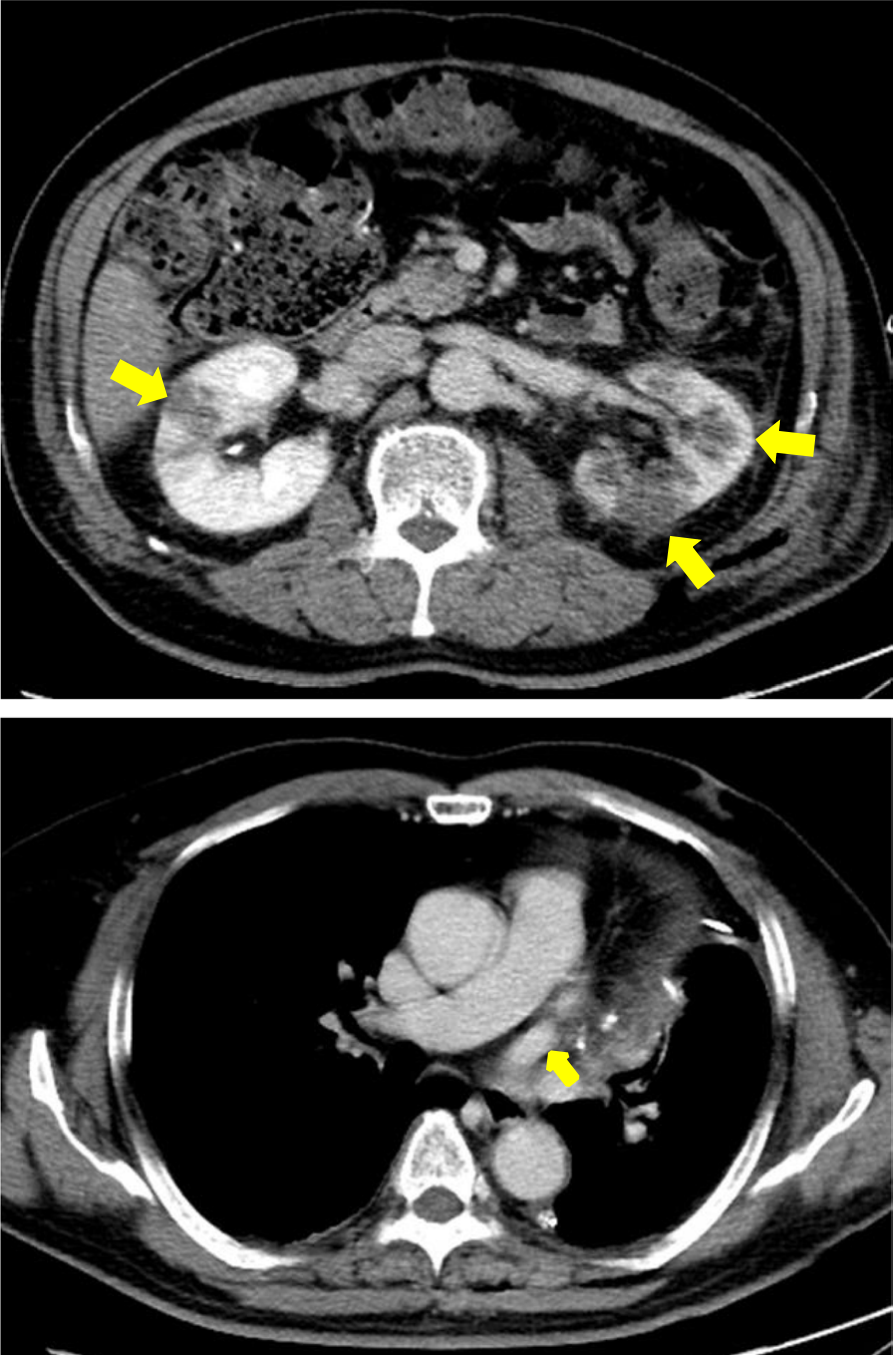
Contrast-enhanced computed tomography showed partial defect of the bilateral kidney and no PVS thrombosis

**Fig. 3:**
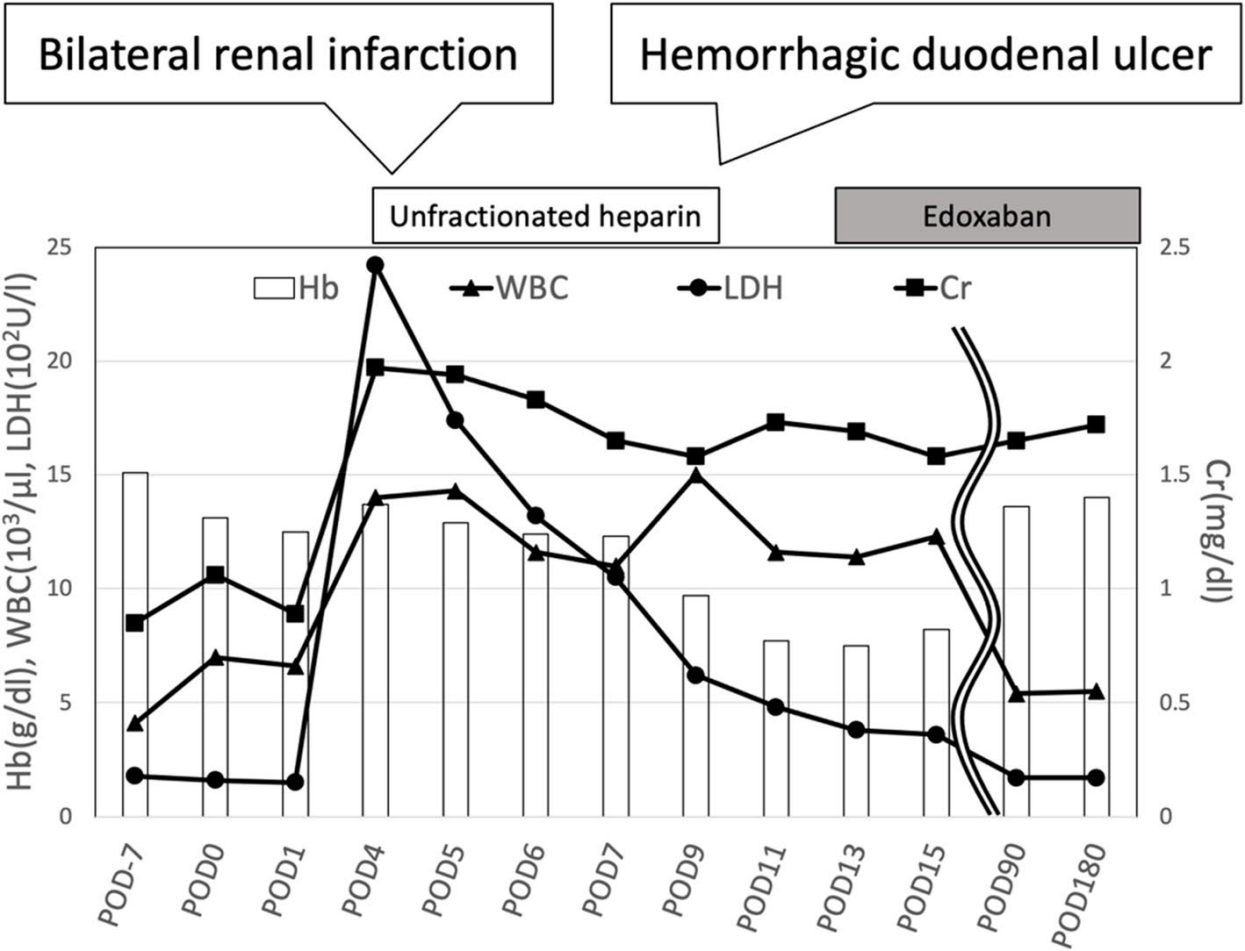
Clinical course. Hb: hemoglobin, WBC: white blood cell count, LDH: lactate dehydrogenase, Cr: serum creatinine, POD: postoperative day

**Fig. 4:**
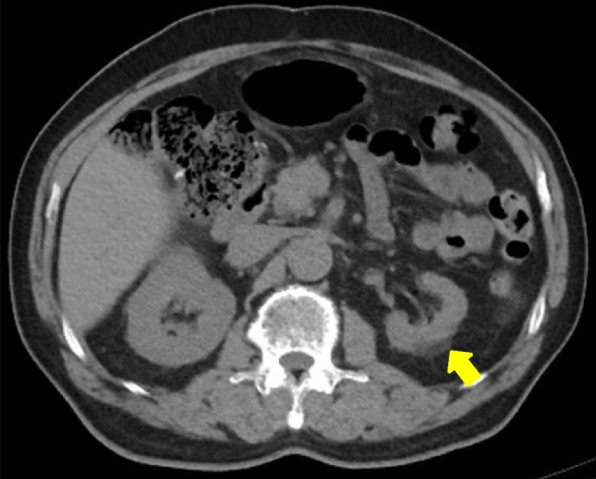
Abdominal computed tomography showed that the scar formation was detected in the area of renal infarction, on 3 months after the operation

A pathological evaluation showed that the maximum diameter of the tumor was 2.6 cm. The histological type was non-keratinizing squamous cell carcinoma, and the final pathological stage was pT1cN0M0 (p-stageIA3). No complications were observed during the operation, and the immediate postoperative period was uneventful until postoperative day (POD) 3. On POD 4, he experienced fever, abdominal pain (not back pain), and nausea, which spontaneously resolved. Laboratory data showed an elevated white blood cell count (14,000/μl), and elevated serum lactate dehydrogenase (2416 IU/l) and creatinine (1.97 mg/dl) concentrations. Contrast-enhanced computed tomography (CECT) from the thorax to the pelvic cavity showed a partial defect of both kidneys (Fig. 2) and no PVS thrombosis. We diagnosed the patient with bilateral renal infarction, and heparin (10,000 IU/day) was initiated. On POD 9, heparin (10,000 IU/day) was temporarily suspended because of hemorrhage from a duodenal ulcer. On POD 13, the patient was asked to continue taking edoxaban 30 mg/day. On POD 16, the patient was discharged from hospital (edoxaban same dose) (Fig. 3). Three months after the operation, the serum creatinine concentration did not show any remarkable elevation compared with the postoperative value (1.97 to 1.7 mg/dl). Computed tomography showed scar formation in the area of renal infarction (Fig. 4). One year after the operation, computed tomography showed no specific changes in the area of renal infarction. In ongoing follow-up, no new infarction has been observed.

## Discussion

Recently, PVS thrombus after LUL has been considered as a possible cause of postoperative systemic embolic morbidities. LUL is considered a risk factor for thrombus formation in the PVS compared with other lobectomies, and it occurs in 11.7–13.5% of cases [1, 3]. Although cerebral infarction after LUL has been previously reported [3–5], reports of bilateral renal infarction after lobectomy are rare. We report a case of acute bilateral renal infarction after LUL.

The development of thrombosis in this case was thought to be related to enhanced coagulation caused by carcinoma (lung, colon, and liver metastasis of the colon). Excessive coagulation associated with cancer is known as Trousseau’s syndrome because Trousseau et al. showed that patients with malignant disease have a potential risk of thrombosis [6, 7]. There are multiple overlapping and interacting mechanisms of Trousseau’s syndrome, such as mucin, tissue factor, cysteine proteinase, and inflammatory cytokines that activate the endothelium and platelet adhesion molecules.

Patients with cancer have an increased risk of arterial thromboembolism even before the diagnosis of cancer, and its risk is greater in more advanced stages of cancer [8]. Among patients with various types of cancer, patients with lung cancer have the greatest excess risk of arterial thromboembolism, with a 3-month cumulative incidence of 6.5% since diagnosis compared with 1.2% in healthy control patients (without any cancer) [8]. The risk of arterial thromboembolism in Trousseau’s syndrome remains unknown. However, the clear association between the cancer stage and risk of arterial thromboembolism suggests a biological gradient between cancer activity and the risk of arterial thromboembolism [8].

In this case, no obvious abnormalities, such as deep vein thrombosis, valvular disease, and arrhythmia, were found in any tests from postoperatively to post-thrombosis onset. There were also no obvious complications in the postoperative course.

Pulmonary vein (PV) thrombosis after LUL might also be developed in our patient. This might have been due to the anatomical fact that the stump of the LSPV is usually longer than that of other PVs [1]. Blood flow stasis in a long stump might predispose LSPV to form a thrombus. To prevent thrombus formation in the PVS, a surgeon proposed transecting the PV inside the pericardium [9]. However, this transection procedure inside the pericardium would increase the operation time and increase the risk of bleeding during surgery. In addition, transecting the PV inside the pericardium is technically difficult in the thoracoscopic setting [10]. Nakano et al. [11] recently reported a new surgical approach to prevent thrombus formation, in which the PV is ligated at the pericardial reflection before transecting the PV. This might be a surgical approach that can be used for a reference.

Table 1 summarizes cases of renal infarction after pulmonary resection reported in the literature [2, 10, 12–19]. To date, all cases of renal infarction after pulmonary resection have primarily occurred in patients who underwent LUL, and PVS thrombosis was observed in five (50%) patients. This finding suggests that PVS thrombus may also be involved in arterial thrombosis other than cerebral infarction. Renal infarction after pulmonary resection occurred in nearly all patients within POD 5. Most of the onset of thrombus was found in the early phase after pulmonary lobectomy or with further intervention (i.e., 80% within POD 4). In this case, treatment could be started as soon as possible after the diagnosis was confirmed, and organ atrophy in the distant phase was mild.

**Table 1:**
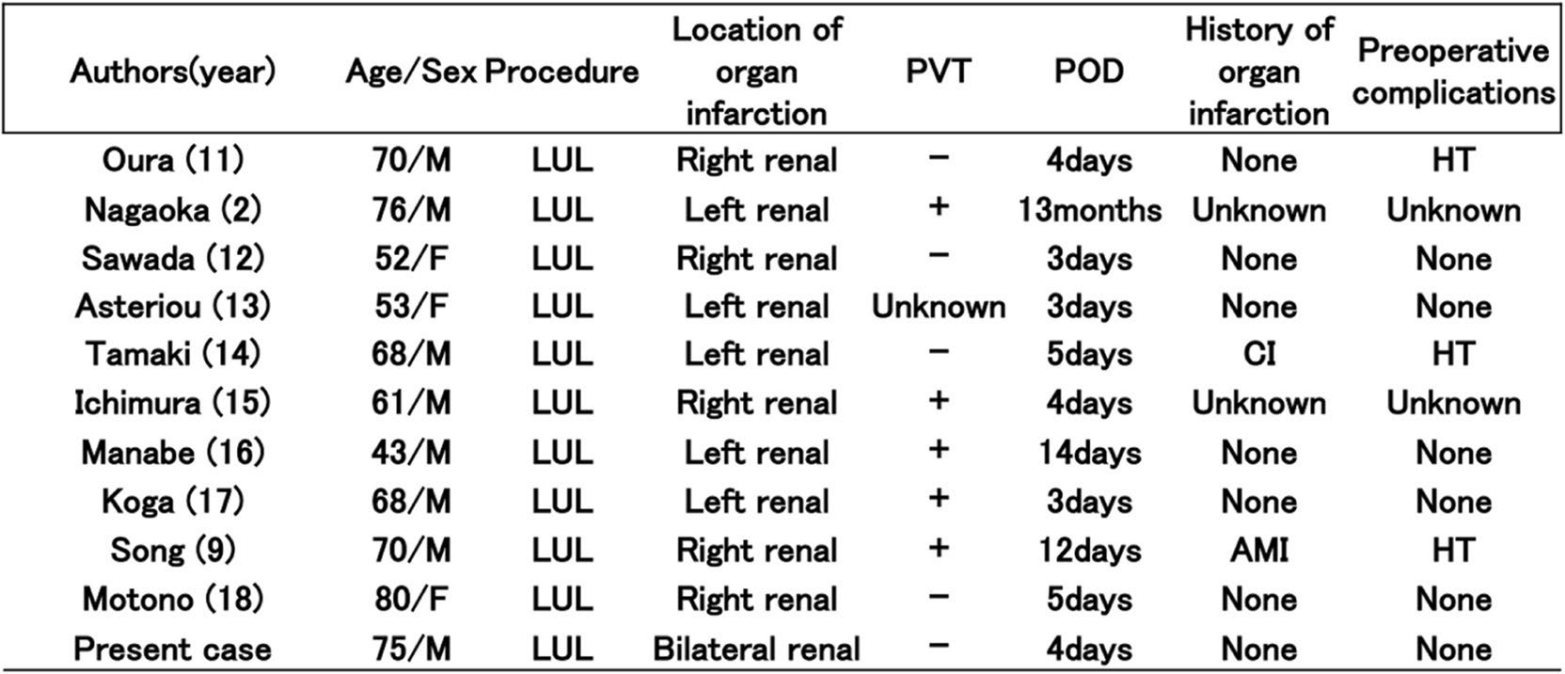
Cases of renal infarction after pulmonary resection reported in the literature

*PVT* Pulmonary vein thrombus, *POD* Postoperative day, *LUL* Left upper lobectomy, *CI* Cerebral infarction, *AMI* Acute myocardial infarction, *HT* Hypertension

However, because of the lethality and serious complications of the formation of thromboembolism after pulmonary resection, new evidence for postoperative management is required. The safety and usefulness of performing postoperative anticoagulant therapy and early CECT depending on the risk should be considered. Abdominal infarction is a rare condition in which the diagnosis is difficult to confirm. Additionally, because there is a risk of bleeding in the early postoperative period, the timing of initiating treatment with anticoagulant therapy is difficult.

Therefore, if the etiology and risk factors of thrombus formation after LUL are clearly identified at this time, we could effectively provide anticoagulant therapy to prevent the postoperative renal infarction as much as possible. To resolve these issues and possibly prevent postoperative renal infarction after general thoracic surgery, the presence of PVS thrombus should be evaluated by performing postoperative CECT for patients who undergo LUL. Furthermore, a survey should be performed in the early phase after LUL because postoperative renal infarction usually occurs immediately after pulmonary resection. The early detection of a thrombus may contribute to the protection of postoperative renal function. Therefore, a multicenter clinical study should be performed to determine the frequency, timing, and conditions favoring the formation of PVS thrombus using postoperative CECT.

In conclusion, bilateral renal infarction after LUL is a rare and serious condition that should be diagnosed early, if nonspecific symptoms appear after LUL, early CECT should be considered to detect acute arterial thrombosis, including renal infarction. Special attention should be paid to postoperative management after LUL. In addition, only for patients who found to have PVS thrombus on CECT should be started to anticoagulation early.

## Data Availability

Data sharing is not applicable to this article as no datasets were generated or analyzed during the current study.

## Abbreviations

CECT: Contrast-enhanced computed tomography
Cre: Creatinine
Hb: Hemoglobin
LDH: Lactate dehydrogenase
LUL: Left upper lobectomy
LSPV: Left superior pulmonary vein
POD: Postoperative day
PV: Pulmonary vein
PVS: Pulmonary vein stump
WBC: White blood cell count
